# Electronic Couplings for Triplet–Triplet Annihilation Upconversion in Crystal Rubrene

**DOI:** 10.1021/acs.jctc.4c00185

**Published:** 2024-05-14

**Authors:** Aitor Diaz-Andres, Claire Tonnelé, David Casanova

**Affiliations:** † Donostia International Physics Center (DIPC), Donostia 20018, Euskadi, Spain; ‡ IKERBASQUE, Basque Foundation for Science, Bilbao 48009, Euskadi, Spain

## Abstract

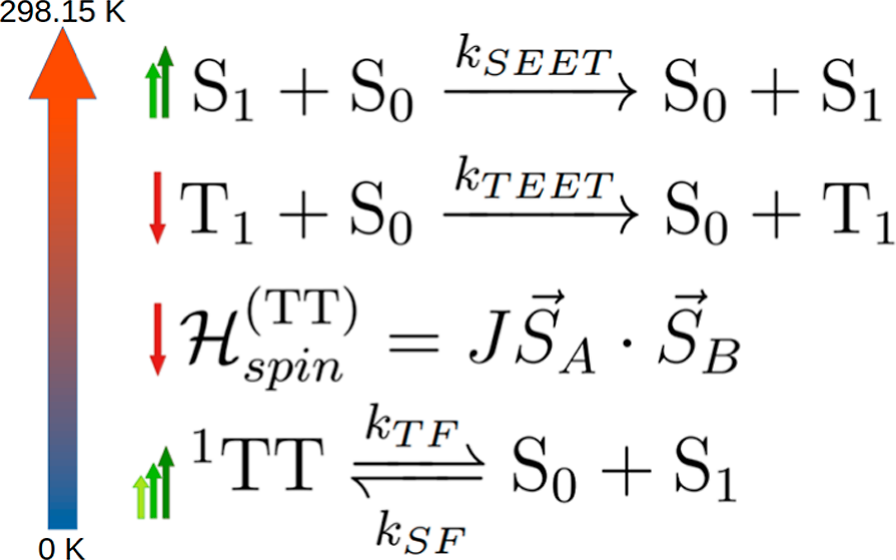

Triplet–triplet annihilation photon upconversion (TTA-UC) is a process able to repackage two low-frequency photons into light of higher energy. This transformation is typically orchestrated by the electronic degrees of freedom within organic compounds possessing suitable singlet and triplet energies and electronic couplings. In this work, we propose a computational protocol for the assessment of electronic couplings crucial to TTA-UC in molecular materials and apply it to the study of crystal rubrene. Our methodology integrates sophisticated yet computationally affordable approaches to quantify couplings in singlet and triplet energy transfer, the binding of triplet pairs, and the fusion to the singlet exciton. Of particular significance is the role played by charge-transfer states along the *b*-axis of rubrene crystal, acting as both partial quenchers of singlet energy transfer and mediators of triplet fusion. Our calculations identify the π-stacking direction as holding notable triplet energy transfer couplings, consistent with the experimentally observed anisotropic exciton diffusion. Finally, we have characterized the impact of thermally induced structural distortions, revealing their key role in the viability of triplet fusion and singlet fission. We posit that our approaches are transferable to a broad spectrum of organic molecular materials, offering a feasible means to quantify electronic couplings.

## Introduction

Triplet–triplet annihilation (TTA) was characterized for the first time more than 60 years ago in solutions of anthracene and phenanthrene.^1^ In the early 2000s, TTA was suggested as a potential strategy to enhance the efficiency of photovoltaic devices by up-converting low-energy solar photons, holding the promise to surmount the inherent limitations of single-junction solar cells.^2,3^ Beyond the field of photovoltaics, materials promoting TTA-based photon upconversion (TTA-UC) have found applications for improving the performance of organic light-emitting diodes (OLEDs),^4^ as anti-Stokes fluorescence labels for bioimaging and drug-targeting,^5^ or in optogenetics.^6^ Driven by these diverse potential applications, the TTA-UC photophysical process has gained a lot of interest, leading to remarkable progress over the last two decades.^7^ These advances have not only deepened our comprehension of fundamental aspects but have also catalyzed the design of solid-state TTA-UC systems,^8,9^ the development of efficient photosensitizers,^10^ the identification of critical parameters in TTA-UC,^11^ the development of organic–inorganic hybrid interfaces for triplet energy transfer,^12^ and the improvement of photovoltaic efficiencies.^13^

Rubrene, i.e., 5,6,11,12-tetraphenyltetracene, is a paradigmatic and benchmark compound in the field of organic optoelectronics, known for its performance in OLEDs and organic field-effect transistors. Notably, as the energy of the triplet state in rubrene is close to half that of the lowest excited singlet,^14−17^ the fusion of two triplets to form a singlet state is energetically allowed. Indeed, TTA-UC has been experimentally detected in rubrene crystals,^18,19^ amorphous rubrene,^20−22^ and in solution as well.^7^ In addition to adequate singlet and triplet energies, a photophysical process such as TTA-UC needs to outcompete potential deactivation channels to take place. Since the rate of photophysical reactions is directly related to the strength of the interaction between initial and final states, electronic couplings are key to understanding the viability and performance of processes in molecular materials, e.g., molecular crystals and thin films. The characterization of electronic couplings has been a crucial element to uncover the mechanisms responsible for the markedly anisotropic hole and exciton transport in crystal rubrene^23−27^ and to shed light on the intricacies of singlet fission (SF) in crystal and amorphous rubrene.^28^ Hence, intermolecular interactions might be very sensitive to structural changes. Therefore, it is mandatory to take into consideration thermal fluctuations in order to evaluate electronic couplings at finite temperature. This aspect holds crucial importance in rubrene materials as it has been shown that charge mobility in rubrene crystals is temperature-dependent.^29−33^

Several photophysical reactions are typically involved in the solid state TTA-UC phenomenon. Triplet excitons, generated either through photon absorption or charge recombination, can undergo triplet exciton energy transfer (TEET) to neighboring molecules (eq 1). The collision of two triplet excitons, facilitated by their extended lifetime,^34^ enables the formation of an intermediate triplet-pair (multiexcitonic) state,^35,36^ denoted as ^*l*^TT (triplet–triplet formation, TTF in eq 2). This state possesses an overall spin (*l*), resulting from the coupling of two *S* = 1 states, with *l* taking values of 1 (singlet), 3 (triplet), or 5 (quintet). The formation and dissociation of the coherent triplet-pair multiexcitonic state has actually been confirmed experimentally in rubrene single crystals by the presence of periodic modulations in the photoluminescence dynamics under a magnetic field.^37^ The ^1^TT state can transition to the first excited singlet state (S_1_) via triplet fusion (TF), as described in eq 3. The reverse reactions of TTF and TF are referred to as triplet–triplet dissociation (TTD) and SF,^38^ respectively. Finally, in addition to fission back to the triplet-pair state, singlet excitons can diffuse through the system via singlet exciton energy transfer (SEET, eq 4), or decay to the ground state via either radiative or nonradiative processes. There can also be other processes in competition with those in ref (14) such as intersystem crossing from S_1_ to the triplet manifold or molecular degradation through photochemical reactions. Yet, these play only minor roles in crystal rubrene and will thus not be considered here.
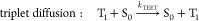
1
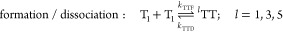
2
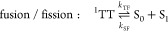
3
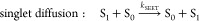
4

The rates of the processes in eqs 1–4 control the properties and efficiency of TTA-UC and are linked to a set of key parameters as expressed by Fermi’s golden rule^39^
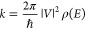
5where *V* represents the electronic coupling between initial and final states, and ρ(*E*) denotes the density of states at energy *E*. The former depends on the specific arrangement of molecules in the extended system, while the latter is generally associated with the energy alignment of states. This alignment can be modeled using various approximations, such as the Marcus^40^ or the Bixon–Jortner^41^ models.

In this work, we conduct a computational investigation into the nature and characteristics of TTA-UC couplings in crystal rubrene. We explore different approaches for the calculation of electronic couplings relevant for the individual processes involved in TTA-UC (eqs 1–4), analyze the role of thermally induced structural distortions, and propose a computational protocol that can be broadly applied to organic molecular materials.

## Computational Details

### Rubrene Crystal Structure

**Figure 1:**
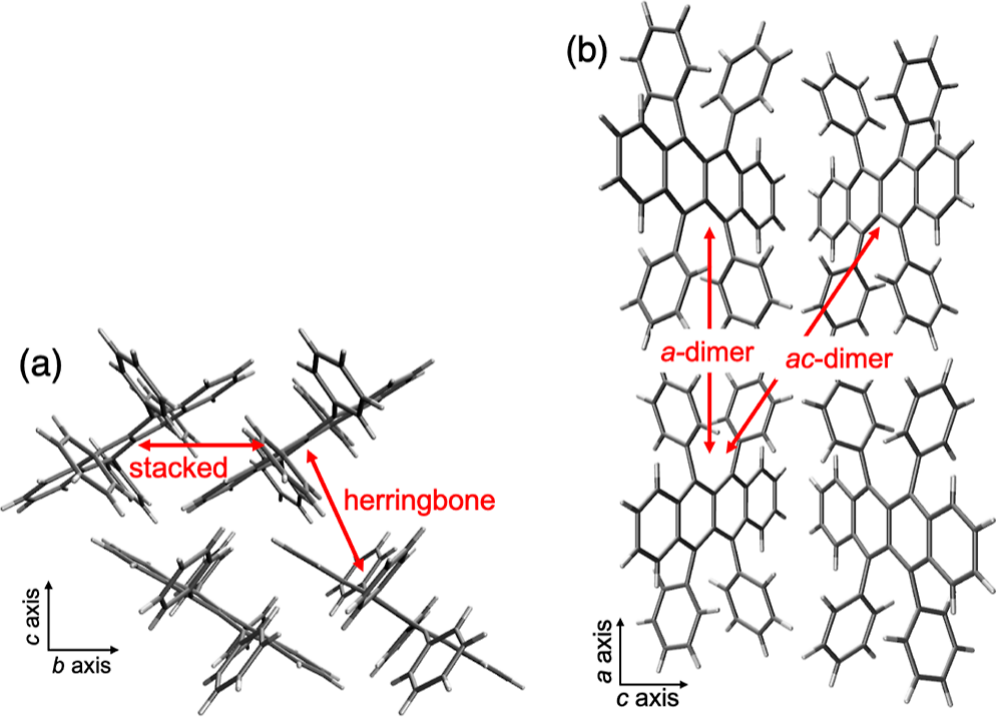
Crystal structure of rubrene along the *bc* (a) and *ac* (b) planes. Red arrows indicate the crystal rubrene dimers studied.

We investigate intermolecular interactions in the four unique first neighbor rubrene dimers within the orthorhombic crystal (Figure 1)^42^ extracted from the Cambridge Structural Database (CSD).^43^ Unique rubrene dimers were selected from the rubrene crystal unit cell, and symmetric representations were removed. All electronic structure calculations were performed on the (frozen) molecular structure from the crystal, i.e., with no further geometry optimizations.

The structural parameters, namely, intermolecular distance and relative orientation, largely dictate intermolecular interactions. In particular, the π-stacked (*b*-axis) and herringbone (*c*-axis) dimers exhibit shorter separations compared to the first neighboring dimer along the *a*-axis (*a*-dimer) and the first neighboring dimer in the *ac*-direction (*ac*-dimer) (Table S1). It is important to note that while in the gas phase or in amorphous films, the rubrene molecule acquires a twisted backbone,^44−46^ noncovalent interactions in the crystal enforce planarity on the tetracene moiety.^47^

### Electronic Structure Calculations

Calculations of the lowest-lying excited states of rubrene molecule and dimers were performed using time-dependent density functional theory (TDDFT) within the Tamm–Dancoff approximation (TDA).^48^ Charge transfer (CT) energies, corresponding to anion–cation configurations of the rubrene dimers, have been computed with constrained DFT (C-DFT)^49^ by imposing the two rubrene molecules of the dimer to hold −1 and +1 charges, respectively, using Becke’s atomic partitioning functions.^50^ All DFT-based calculations were carried out with the rCAM-B3LYP energy functional.^51^ In order to include the overall spin singlet multiexcitonic triplet-pair state (^1^TT) in our study, we employed the restricted active space configuration interaction (RASCI) method with and without one hole and one particle contributions,^52−55^ with the Hartree–Fock (HF) wave function as the reference configuration, considering a RAS2 space with four electrons in four orbitals, and with the cc-pVDZ basis set (Table 1). This method was also used to compute singlet, triplet, and quintet excited states from a singlet ground state reference. We recognize that other strategies could also be applied to the challenging characterization of the ^1^TT state. In particular, a single spin-flip (1-SF) strategy was proposed, compatible with the limitations of linear response TDDFT.^56^ Triplet-pair binding energies in rubrene dimers were computed with the RASCI method within the one hole and one particle approximation, which has proven successful in characterizing TT states in various SF organic compounds.^57−64^ A comparative analysis of the dependence of the computed electronic couplings with the employed level of correlation and basis set can be found in Table S2.

**Table 1:** Electronic Couplings (in meV) for SEET and TEET Processes in the Four First-Neighbor Crystal Dimers Computed at the RASCI/cc-pVDZ Level^a^

dimer	dip–dip	HEG	FED	Boys(2)	Boys(5)
SEET					
stacked	50.6	13.1	21.3	17.7	26.8 (*48.0*)
herringbone	37.0	33.3	34.9	31.8	31.8 (*31.7*)
*a*-dimer	13.9	12.7	13.0	12.5	12.5 (*12.5*)
*ac*-dimer	7.2	7.1	7.3	7.0	7.0 (*7.0*)
TEET					
stacked		11.4	11.2	12.9	
herringbone		4.1	0.2	0.4	
*a*-dimer		0.0	0.0	0.0	
*ac*-dimer		0.0	0.0	0.0	

a The value in parentheses for Boys diabatization calculations indicates the number of states considered in the adiabatic–diabatic transformation. Boys(5) values in parentheses and italics correspond to first-order (direct) exciton coupling. We have disregarded the use of the Boys(5) scheme for the computation of TEET due to the large energy gap between the lowest triplet and CT states.

All electronic structure calculation were performed with the cc-pVDZ basis set and using the Q-Chem program package.^65^ Molecular orbitals were analyzed with the IQmol molecular viewer.^66^

### Molecular Dynamics

Molecular dynamics simulations of the bulk rubrene crystal were carried out with the NAMD program,^67^ using a force field specially derived for rubrene.^33^ Torsional parameters for the dihedral angle between the tetracene and phenyl moieties were derived from DFT calculations at the PBE0/def2-TZVP level, while atomic charges were obtained at the PBE0/TZVP level using benzene as the implicit solvent. Additionally, the carbon and hydrogens atoms Lennard-Jones parameters were tuned in a trial-and-error procedure running simulations at both *T* = 100 K and *T* = 300 K at ambient pressure, systematically comparing simulated and experimental crystal lattice parameters *a*, *b*, and *c*, and cell volume. A quasi cubic sample containing 256 rubrene molecules was first equilibrated in the *NpT* ensemble (*p* = 1 atm) at 298.15 K for 9 ns before a 1 ns production run, from which 20 frames were extracted at regular time intervals. Pressure and temperature controls were achieved using the Berendsen’s barostat and the velocity scaling thermostat, respectively.

### Exciton Energy Transfer

Firstly, we consider the couplings related to exciton energy transfer, both in the singlet and triplet manifold. For that, we explore the classical dipole–dipole interaction, the half energy gap (HEG) rule, the fragment excitation difference (FED),^68^ and Boys exciton diabatization of excited states.^69^

Interchromophore energy transfer triggers exciton diffusion in organic molecular solids. In the long-range limit, the overlap between the electronic wave functions of the excited donor and the ground state acceptor molecular sites vanishes.^70,71^ Hence, their interaction can be described as a purely electrostatic dipole–dipole interaction^72^
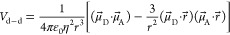
6where  are the transition dipole moments of the donor (D) and acceptor (A) chromophores, *r⃗* is the intermolecular distance vector, and η is the medium refractive index. In the present study, we compute *V*_d–d_ under vacuum (η = 1). The point dipole approximation is successfully applied for intermolecular distances of a few nm or more, as in Förster resonance energy transfer, but it is no longer valid for shorter separations as molecular size effects and orbital overlap become important.^73^ Moreover, eq 6 cannot describe the energy transfer of dark states, e.g., triplet states. In those cases, intermolecular excitonic couplings between a pair of equivalent molecules can be heuristically estimated by the HEG rule
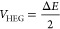
7where Δ*E* is the energy difference between the states involved as computed in the dimer. This rule provides a rough estimate of the strength of the interaction at stake in excitation energy transfer processes. More sophisticated approaches are based on the idea of diabatic states. Under the two-state approximation, these are built as linear combinations of eigenstates of the dimer. In FED, couplings are obtained by assuming a maximum excitation difference (Δ*x*_*mn*_)
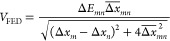
8where Δ*E*_*mn*_ is the energy difference between eigenstates *m* and *n*, Δ*x*_*mn*_ is the donor–acceptor difference of the sum of attachment and detachment densities for the transition between *m* and *n*, and the over bar indicates symmetrized quantities.

The two-state treatment can be expanded to accommodate the transformation of multiple adiabatic to diabatic states. However, the transformation between adiabatic and diabatic basis lacks a universal procedure.^74,75^ Various strategies have been proposed, with some relying on orbital localization schemes, such as the Boys method. In this approach, diabatic states are derived by maximizing the charge separation between them
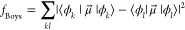
9where {ϕ_*i*_} are the Boys diabatic states. Here, the interstate couplings can be directly evaluated as the off-diagonal elements of the electronic Hamiltonian in the diabatic basis. Moreover, the multistate treatment in diabatization schemes allows the coupling of initial and final states through intermediate diabats (mediators). In terms of perturbation theory, electronic couplings can be expressed as the sum of direct (first-order) and indirect (second-order) contributions^58^
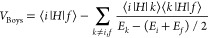
10

Exciton energy transfer couplings (SEET and TEET) between first neighbor dimers of the crystal rubrene were computed using eqs 6 to 10 with the RASCI/cc-pVDZ method (Table 1). Electronic couplings at the rCAM-B3LYP/cc-pVDZ level can be found in the Supporting Information (Table S3).

The SEET values obtained with the different approaches, typically on the order of tens of meV, are in very good agreement with each other, except for those predicted by the point dipole approach for the dimers with short intermolecular separation (stacked and herringbone dimers), which are considerably larger. This discrepancy is somehow expected as the point dipole approach breaks down at short distances due to the omission of molecular size effects and intermolecular orbital overlaps. On the other hand, dipole–dipole couplings for *a*-dimer and *ac*-dimer are much closer to those from electronic structure calculations.

Interestingly, for all four dimers, the methods that take electronic structure considerations into account, namely, HEG, FED, and Boys(*n*) diabatization (with *n* the number of states considered), produce nearly identical results, with the herringbone dimer exhibiting the largest SEET interaction. The couplings computed with HEG, FED, and Boys(2) account for exciton coupling between localized singlets, that is, interaction between S_1_S_0_ and S_0_S_1_ configurations, but they also implicitly contain potential anion–cation (AC) and cation–anion (CA) contributions partially mixed in the two lowest singlet eigenstates. In order to explore the role of CT states in SEET couplings, we expand the dimension of our diabatization scheme to five states [Boys(5) in Table 1], adding the two lowest singlets with strong CT character and the multiexcitonic state mostly corresponding to the triplet-pair state (^1^TT). This approach allows us to describe the couplings in terms of direct (first-order) and mediated (second-order) contributions (eq 10). The results clearly indicate that the direct exciton coupling dominates in all dimers except for the stacked dimer, for which the CT-mediated terms partially cancel the direct interaction. Therefore, despite the favorable intermolecular disposition for the exciton interaction in the stacked dimer [as indicated by the dipole–dipole interaction and the Boys(5) direct coupling], the mixing of local excitons with charge-separated terms notably diminishes the total coupling. We thus conclude that, in this case, CT excitations are detrimental for the singlet exciton diffusion in crystal rubrene, specifically along the *b*-axis.

The dark character of triplet excitons (zero transition dipole moment) prevents the use of dipole–dipole approach for TEET. Moreover, here we do not consider the diabatization scheme with five states since ^3^CT (spin-triplet CT states) and ^3^TT are energetically much higher than the monomeric triplet (T_1_S_0_ and S_0_T_1_), and state mixing can be safely disregarded. TEET couplings are notably smaller compared to those obtained for singlet excitons. Additionally, they manifest a substantial decline with increasing intermolecular distance, in accordance with the exponential decay pattern outlined by the Dexter energy transfer rate formula.^76^ The strongest TEET interactions are along the *b*-axis (11–13 meV) since the π–π overlap is favored in the coplanar stacked dimer. On the other hand, triplet exciton diffusion is predicted to be very inefficient on the *ac* crystal plane and specially along the *a*-direction.

It is worth noticing that the SEET results in Table 1 seem to be in contradiction with experimental measurements, which indicate that exciton diffusion in the crystal is preferred along the molecular stacking direction (*b*-axis).^24,77^ However, it has been shown that the anisotropic exciton diffusion in rubrene single crystals is driven by triplet exciton transport, available by singlet–triplet interconversion via singlet fission and triplet fusion annihilation processes.^27,78^ In fact, this is in agreement with our TEET results, indicating the tendency of triplet excitons to move along axis *b*.

### Triplet-Pair State

The multiexcitonic triplet-pair state plays a pivotal role in the TTA-UC process within organic condensed phases, exemplified by crystalline rubrene. When two nongeminate triplets encounter each other, they can form an overall state that delocalizes across two interacting molecules. The electronic structure and properties of the triplet-pair state have been extensively studied, primarily in the context of singlet fission.^35,36,58^ Recently, the role of triplet-pair multiplets in the TTA-UC dynamics in solid rubrene has been thoroughly investigated.^21^

The TT manifold can be effectively modeled using spin Hamiltonians. In the absence of an external magnetic field (i.e., no Zeeman interaction) and neglecting relativistic effects, the spin Hamiltonian simplifies to an intertriplet exchange interaction (*J* in eq 11)
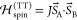
11where subindices A and B refer to interacting centers (rubrene molecules in this case). This approach holds in the regime of strong exchange coupling, which is well-justified in tetracene-based molecular materials as exchange interactions are significantly larger than both intra- and intertriplet–triplet zero-field splittings.^79−82^

The eigenstates of the spin Hamiltonian in eq 11 correspond to nine spin-adapted states (eigenstates of ), resulting from the coupling of the two triplets.^21^ Therefore, we can correlate their eigenenergies with those obtained from ab initio calculations. Specifically, we employ the energy difference between the high (quintet) and low (singlet) solutions to quantify *J*
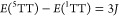
12

Evaluating *J* via eq 12 necessitates a balanced computation of ^1^TT and ^5^TT states. The high-spin state (*M*_S_ = ± 2) is predominantly governed by a single configuration, making it amenable to accurate computation using single-reference post-HF methods, such as MP2 or CCSD, or even Kohn–Sham approximations. In contrast, the calculation of ^1^TT is considerably more challenging due to its pronounced spin correlation.^83^ Additionally, the pristine ^1^TT state can exhibit mixing with other configurations, which may account for approximately 10% of the wave function in organic crystals.^84^ Various strategies have been explored to efficiently compute this doubly excited singlet state.^85^ However, the two-electron transition nature from the ground state prevents the applicability of standard excited state methods.^86^ For instance, (linear response) TDDFT with the adiabatic approximation is unable to capture states with double excitation character and hence cannot be used to describe the ^1^TT state.^87−91^ Accurate post-HF approaches, such as the equation-of-motion coupled cluster singles and doubles (EOM-CCSD) method, while explicitly incorporating higher order excitations, can be highly inaccurate for electronic states dominated by two-electron excitation character.^92−94^ Furthermore, the computational demands of these methods make their application challenging for medium to large systems, such as rubrene dimers.

**Figure 2:**
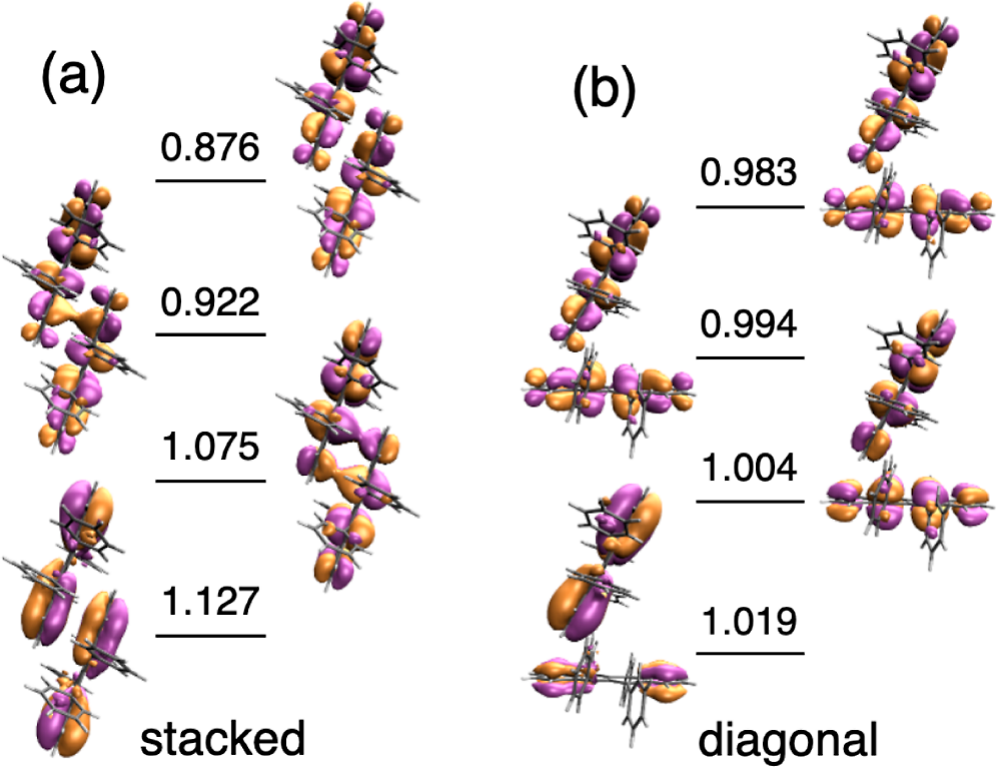
Frontier natural orbitals and their electron occupancies for the ^1^TT state of the stacked and herringbone first-neighbor rubrene crystal dimers computed at the RASCI/cc-pVDZ level.

Our results at the RASCI level indicate a substantial exchange interaction favoring the spin-singlet state for the π-stacked rubrene dimer, with *J* = 7.5 meV (antiferromagnetic coupling), while the singlet, triplet, and quintet TT states are nearly degenerated (*J* ≈ 0 meV) in the three other dimers. These results can be rationalized by the degree of intermolecular overlap in the natural orbitals of the triplet-pair state (see Figure 2), which is significantly larger in the stacked dimer compared to the others. In other words, the electronic triplet–triplet binding energy is only significant in the π-stacked dimer, for which orbital interaction stabilizes ^1^TT with respect to ^5^TT. This behavior is also manifested by the distribution of natural orbital occupancies of the ^1^TT state, with values slightly departing from four orbitals with a single electron each, which is a sign of (small) electron pairing.

### Triplet Fusion and Singlet Fission

Next, we explore the interactions controlling the spin-allowed transition from ^1^TT to S_1_ in crystal rubrene. It is well-known that the direct coupling between ^1^TT and S_1_ states is, in many cases, very small^62,95^ since it involves a change of two electrons. Within the two-electrons-in-two-orbitals model, i.e., only considering HOMO and LUMO of each chromophore, direct couplings correspond to the difference between two-electron integrals.^96,97^ On the other hand, charge-separated states can strongly interact with both ^1^TT and S_1_, mediating their interconversion through the second-order term in eq 10. Therefore, here we employ the second-order perturbative approach with ^1^TT and S_1_S_0_ as initial and final states, respectively. Electronic states and couplings have been obtained with the Boys diabatization of five excited singlet states computed at the RASCI/cc-pVDZ level, resulting in five diabatic states with pristine S_1_S_0_, S_0_S_1_, AC, CA, and ^1^TT nature, respectively.

Evaluation of the second-order contribution to the total coupling requires diabatic energies. Although RASCI with one-hole and one-particle contributions has shown excellent performance in the characterization of electronic states, it is well understood that the lack of higher order terms can have an important impact on the computed relative energies.^55^ Hence, to mitigate inaccuracies arising from RASCI energies, we employ accurate reference values for the diabatic state energies. In this case, we take experimental energies for the lowest singlet state (*E*(S_1_) = 2.23 eV) and approximate the triplet-pair energy to twice the energy of the lowest triplet [*E*(^1^TT) ≈ 2*E*(T_1_) = 2.28 eV] states.^98^ The relative CT energies computed for isolated dimers have been corrected in order to take into account the surrounding polarizable medium, as prescribed by previous polarization energy calculations in the bulk.^99^ The computed interstate couplings and CT energies are shown in Table 2.

**Table 2:** Electronic Couplings (in meV) Computed at the RASCI and C-DFT Level/cc-pVDZ Level through Boys Diabatization, and CT Energies, *E*(CT) = (*E*(AC) + *E*(CA))/2 (in eV), Computed at the C-DFT Level/cc-pVDZ Level for the Four Crystalline Rubrene Dimers^a^

	stacked	herringbone	*a*-dimer	*ac*-dimer
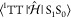	0.0	0.0	0.2	0.0
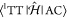	0.0	0.0	2.4	0.6
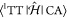	0.0	0.0	2.4	0.6
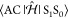	–173.1	–25.7	2.2	0.4
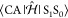	90.7	–8.1	1.6	0.5
*E*(CT)	2.65	3.03	3.87	2.71

a Couplings considering S_0_S_1_ as the final state can be found in Table S4.

Computed direct couplings between initial and final states are very small in all four first neighbor crystal dimers (0.2 meV in a-dimer and below 0.1 meV for the rest). On the other hand, some of the interactions between initial/final diabats and CT states appear to be rather significant. In the two short-distance dimers (stacked and herringbone), the coupling of the localized S_1_ with CT configurations is rather strong and in rather good agreement with previous calculations on the stacked dimer at the multireference second-order perturbation theory level (−175 and 86 meV, respectively),^95^ with the ZINDO approach (67 meV),^28^ and with evaluations of the HOMO–HOMO transfer integral (100–200 meV).^24,25,95,100^ In contrast, the ^1^TT/AC and ^1^TT/CA couplings vanish for the stacked and herringbone dimers. The effectively zero interaction between the spin-singlet triplet-pair configuration and CT states in the π-stacked dimer has been rationalized by orbital symmetry arguments.^62,95,98,101^

Electronic couplings in a-dimer and ac-dimer involving charge-separated terms are ∼2 meV in the former and ∼0.5 meV in the latter, but their contribution to the total coupling is hindered by the larger energy gap between initial/final states and CT terms, especially in the a-dimer. Moreover, the (small) second-order term in these dimers partially cancels out the direct coupling contribution (opposite signs in eq 10).

All in all, total electronic couplings for the interconversion between the triplet-pair state and S_1_ in the crystal dimers are very weak (smaller than 0.1 eV in all cases). These results seem to prevent the fusion/fission process (eq 3) in the rubrene crystal, which is in strong contradiction with experimental evidence of TTA and SF in rubrene crystals.

### Role of Thermal Fluctuations

So far, we have investigated electronic couplings at the frozen rubrene crystal structure, that is, without taking into account any structural dynamic disorder. It has been shown that slight symmetry breaking distortions can produce large fluctuations in intermolecular interactions, and in particular for the one-electron terms,^102,103^ sensibly modifying the couplings obtained for the nondistorted crystal structure.

In the following, we examine the role of thermal fluctuations in the electronic interactions responsible for the singlet and triplet exciton diffusion, triplet–triplet binding, and in the interconversion between the triplet-pair and the singlet exciton (fusion/fission). For that, we employ classical molecular dynamics (MD) simulations to generate a representative sample of thermally distorted structures (details are given in the Supporting Information), for which we compute interstate couplings and CT energies. In the calculation of electronic couplings, we select the most reliable and computationally affordable methods among those used in the previous sections: SEET and TEET with FED for rCAM-B3LYP states, RASCI for the evaluation of triplet–triplet binding and ^1^TT/S_1_ interaction, the latter with (five-state) Boys diabatization scheme. S_1_ and T_1_ diabatic energies have been fixed to the experimental values used above, while CT states have been recomputed for each dimer. Our approach does not take into consideration the role of crystal defects or energetic disorder (variations in site energies), which can be of relevance in actual devices.^104−107^ Moreover, despite that the relative signs of the computed electronic couplings for a given structure are fully consistent, the obtained phase of diabatic wave functions is not fixed to a specific criterion and might change between separate calculations. Therefore, here we analyze unsigned couplings.

The average couplings over 200 structural MD frames at 298 K are shown in Table 3. Median values can be found in Table S6. The SEET average values are slightly larger than those in the frozen crystal structure, especially for the π-stacked dimer, for which small displacements might involve sizable changes in the orbital interactions. Thermal average TEET couplings and triplet–triplet binding energies are very close to the 0 K values. The most impressive changes upon consideration of thermal fluctuations are for the ^1^TT/S_1_ interaction. While the fusion/fission coupling remains below 0.1 meV for the long-distance dimers, it increases to the order of 1 meV in the herringbone dimer and explodes for the stacked dimer (127 meV). This huge enhancement in the *b*-axis mostly emerges from breaking the symmetry of the molecular dimers responsible for the vanishing coupling between ^1^TT and CT configurations in the frozen crystal. These results unequivocally identify thermal fluctuations as being critical for TF and SF photophysical processes (eq 3) in crystal rubrene.

**Table 3:** Electronic Couplings (in meV) Computed for the Crystal Structure (Cryst.) and Averaged over 200 Snapshots from MD (TA: Thermal Average) for SEET, TEET, Triplet–Triplet Binding (*J*), and ^1^TT/S_1_ (TF/SF)

	SEET		TEET		*J*		TF/SF	
dimer	cryst.	TA	cryst.	TA	cryst.	TA	cryst.	TA
stacked	18.2	26.8	7.2	6.9	7.5	6.9	0.0	126.7
herringbone	30.6	34.3	0.3	0.2	0.0	0.1	0.0	1.1
*a*-dimer	17.8	19.2	0.0	0.0	0.0	0.0	0.0	0.0
*ac*-dimer	9.8	10.6	0.0	0.0	0.0	0.1	0.0	0.0

**Figure 3:**
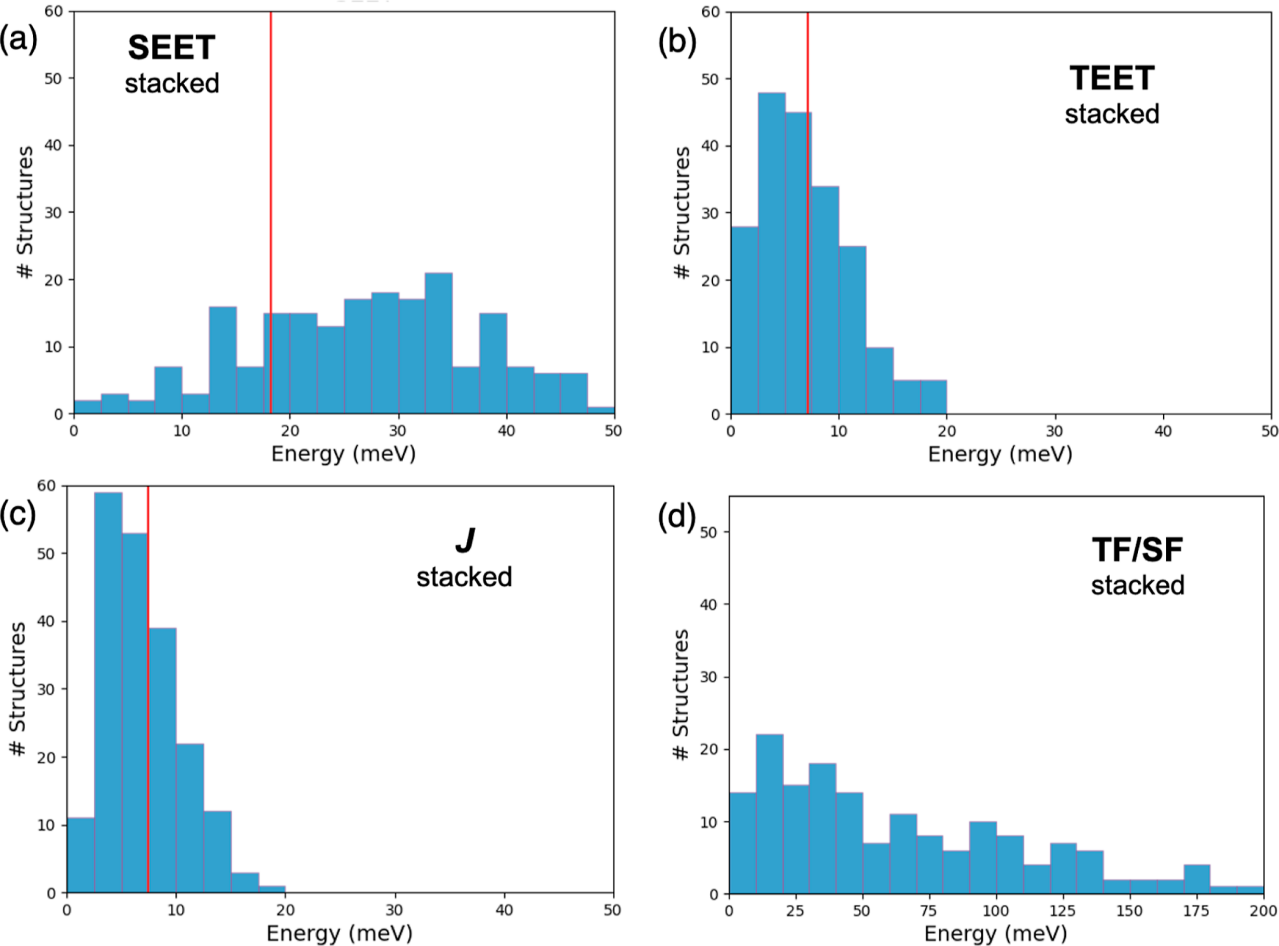
Distribution of absolute electronic couplings (in meV) in π-stacked dimers computed from 200 MD frameworks. (a) SEET, (b) TEET, (c) *J*, and (d) TF/SF. Vertical red lines in (a)-(c) indicate values obtained at 0 K.

Besides the average and median values, it is important to realize the distribution of couplings explored by thermal fluctuations in order to recognize the range of achievable values. Figure 3 shows the set of couplings related to the different studied processes in π-stacked dimers. Distributions for other first-neighbor dimers can be found in the Supporting Information.

SEET couplings are quite symmetrically distributed around the average value, with a small number of arrangements with nearly zero interaction and a few structures reaching values as large as 50 meV. The couplings controlling triplet exciton diffusion present a different distribution, with a rather large density of structures below the average value and a decaying distribution tail of couplings larger than 8 meV. Most of the triplet-pair binding energies of stacked dimers appear within the 0–12 meV range, with few conformations exhibiting stronger triplet–triplet interactions. Finally, although the vast majority of structures exhibit vanishing ^1^TT/S_1_ couplings, the distribution extends to very large interactions (>200 meV). This result indicates that thermally induced motions are very efficient to promote TF or SF in the *b*-direction of the crystal.

**Figure 4:**
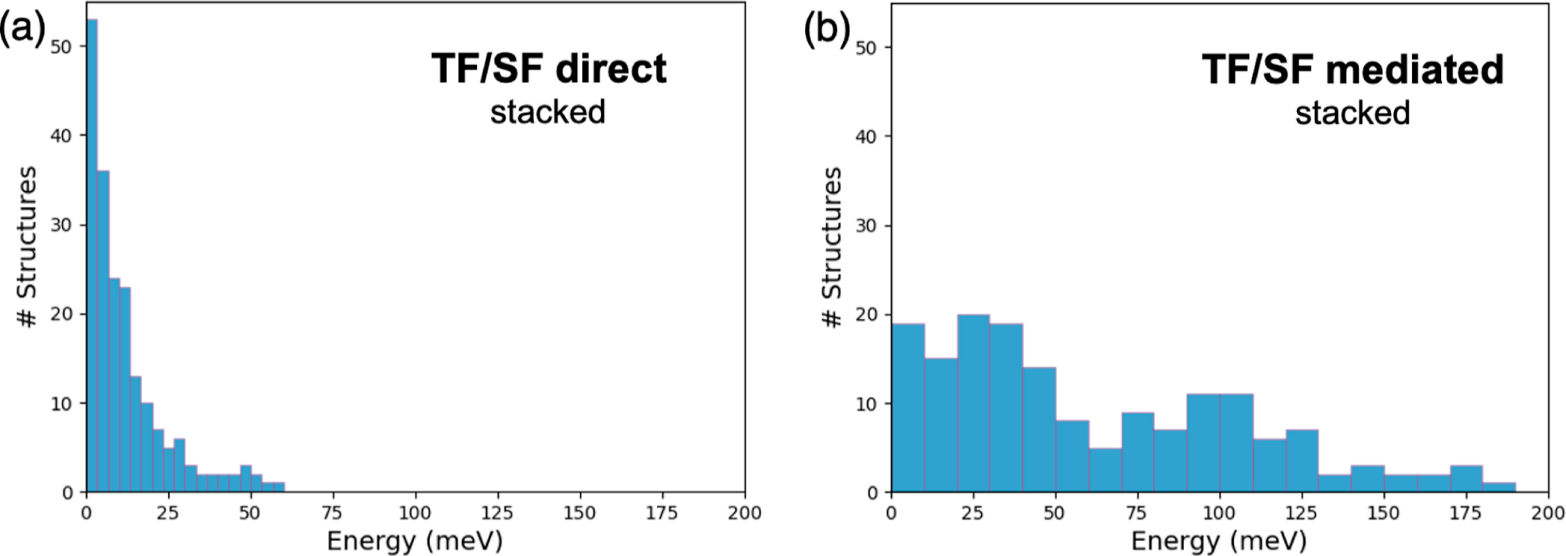
Distribution of absolute TF/SF couplings (in meV) in π-stacked dimers computed from 200 MD frameworks. (a) Direct coupling, (b) second-order (mediated) correction.

To decipher the underlying factors contributing to the notable enhancement in the interaction between S_1_ and ^1^TT states in stacked dimers, we conduct a detailed analysis of both first- and second-order contributions (Figure 4). Examination of the distributions for direct and CT-mediated couplings distinctly reveals that the CT-mediated mechanism predominantly accounts for the large TF/SF couplings observed in numerous MD π-stacked dimers. While the direct contributions may not reach the same magnitude as the second-order terms, a substantial number of sampled structures exhibit considerable first-order interactions, with many exceeding 10 meV. This observation suggests that there are configurations in which the direct pathway plays a significant role.

## Conclusions

In summary, we have described and applied a set of quite sophisticated but computationally affordable electronic structure methods for the evaluation of electronic couplings in TTA-UC in molecular materials. Our strategy provides a unified multielectron approach to account for the complexity of the studied states beyond the one-electron picture. The methodology can be used to identify potential mechanisms by quantifying the role of different diabatic states, such as CT configurations and multiexcitonic states. The low computational cost of the designed protocol allows for the fast evaluation of different couplings for a large number of molecular arrangements, as those required in the study of thermal fluctuations. Despite that here we direct our investigation to TTA-UC in rubrene single crystal, we believe that the validity of these approaches can be extrapolated to a large variety of organic molecular crystals, and that it represents a feasible and yet accurate approach to quantify electronic couplings.

TEET couplings in crystal rubrene are notably stronger along the *b*-axis, in agreement with experimental exciton diffusion measurements. Similarly, triplet–triplet binding energy is significant for π-stacked molecules due to the interaction of π-orbitals of neighboring molecules, while vanishing in the other directions. At 0 K, TF/SF couplings diminish as the direct coupling between ^1^TT and S_1_ is extremely weak, and the interaction of the triplet-pair with CT states is symmetry forbidden. Temperature-induced symmetry-breaking distortions activate TF/SF processes predominantly through the CT-mediated mechanism. These results clearly evidence the crucial role of thermal fluctuations in comprehending the photophysics and exciton transport in crystal rubrene.

## Supporting Information Available

Intermolecular distances between first neighboring rubrene molecules in the crystal structure and additional results for the different electronic couplings in the crystal and along MD simulations (PDF)
