# Supplementary material for: Electronic Couplings for Triplet–Triplet Annihilation Upconversion in Crystal Rubrene
Source: J Chem Theory Comput. 2024 May 14;20(10):4288–97. doi: 10.1021/acs.jctc.4c00185 (PMC11137828; doi:10.1021/acs.jctc.4c00185)
Supplement: Supplementary file 1 — ct4c00185_si_001.pdf [file ct4c00185_si_001.pdf]

Supporting Information: Electronic couplings for triplet-triplet annihilation upconversion in crystal rubrene

Aitor Diaz-Andres,† Claire Tonnelé,†,‡ and David Casanova*,†,‡

†*Donostia International Physics Center (DIPC), 20018 Donostia, Euskadi, Spain*

‡*IKERBASQUE, Basque Foundation for Science, 48009 Bilbao, Euskadi, Spain*

E-mail: david.casanova@dipc.org

Contents

1 Crystal Structure **S2**

2 Couplings and electronic structure method **S2**

3 Singlet and Triplet energy transfer coupling **S3**

4 Triplet-triplet fusion coupling **S4**

5 Triplet state energies **S4**

6 Thermal fluctuations **S4**

7 Structure - coupling analysis **S6**

1 Crystal Structure

Table S1: Intermolecular distances (in Å) between the molecular center of masses for the four unique first neighbor dimers in the orthorhombic crystal structure of rubrene [Acta Crystallogr., Sect. B: Struct. Sci., 2006, 62, 330-334].

dimer	distance
stacked	7.17
herringbone	7.96
a-dimer	13.86
ac-dimer	15.16

2 Couplings and electronic structure method

Table S2: Electronic couplings (in meV) computed for the crystal stacked structure for SEET, TEET, triplet-triplet binding (J) and ^{1}TT/S$_1$ (TF/SF) computed with RASCI with different RAS2 spaces, with and without hole and particle excitations (h, p), and with different basis sets.

			SEET				
RAS2	(h, p)	basis	FED	Boys(5)	TEET	J	TF/SF
$(4, 4)$	no	6-31G	22.7	15.6 (*52.6*)	11.9	1.2	0.0
$(4, 4)$	yes	6-31G	18.5	0.5 (*69.4*)	10.3	6.8	0.0
$(4, 4)$	no	cc-pVDZ	21.3	26.8 (*48.0*)	12.2	1.6	0.0
$(4, 4)$	yes	cc-pVDZ	13.0	15.2 (*65.7*)	11.4	7.5	0.0
$(6, 6)$	no	cc-pVDZ	43.7	0.6 (*76.3*)	9.3	1.7	0.0

The use of (h, p) has almost no impact on the TEET couplings and systematically diminish SEET interactions, although the results remain semi-quantitatively similar to those obtained without (h, p) terms. On the other hand, the presence of (h, p) contributions is rather important in the relative stabilization of the ^{1}TT state with respect to ^{5}TT, as hole and particle terms in the minimal $(4, 4)$ RAS2 space preferentially mix with the singlet triplet-pair state and are decisive in the electronic binding of the two triplets.

The SEET Boys(5) coupling at the RAS(4,4)/6-31G (with hole and particle) and RAS(6,6)/cc-pVDZ (without hole and particle) are very small (< 1 meV). This result can be related to

the inability of the diabatization scheme to produce diabatic states with the pristine local or CT character in these cases.

Therefore, considering the reliability of the methods and their computational cost, we decide to perform RASCI calculations of SEET, TEET and TF/SF with a $(4, 4)$ RAS2 space without *hole* and *particle* excitations. Only for triplet-triplet binding energies we include the (h, p) terms. Although results with the 6-31G basis do not present major discrepancies, we prefer to use the larger cc-pVDZ basis set, as we anticipate that polarization functions might be important to describe some molecular arrangements.

3 Singlet and Triplet energy transfer coupling

Table S3: Electronic couplings (in meV) for SEET and TEET process in the four first-neighbor crystal dimers computed at the rCAM-B3LYP/cc-pVDZ level.

dimer	dip-dip	HEG	FED	Boys(2)
		SEET		
stacked	60.8	13.3	18.2	18.2
herringbone	44.4	31.0	30.6	30.5
a-dimer	15.1	17.8	17.8	17.8
ac-dimer	8.6	9.8	9.8	9.8
		TEET		
stacked	-	7.2	7.2	7.2
herringbone	-	3.0	0.3	0.4
a-dimer	-	0.0	0.0	0.0
ac-dimer	-	0.0	0.0	0.0

4 Triplet-triplet fusion coupling

Table S4: Electronic couplings (in meV) computed at the RASCI and C-DFT level/cc-pVDZ level through Boys diabatization, and CT energies, $E(\mathrm{CT}) = (E(\mathrm{AC}) + E(\mathrm{CA}))/2$ (in eV), computed at the C-DFT level/cc-pVDZ level for the four crystalline rubrene dimers.

	stacked	herringbone	*a*-dimer	*ac*-dimer
$\langle {}^1\mathrm{TT}\vert\hat{\mathcal{H}}\vert \mathrm{S_0S_1}\rangle$	0.0	0.0	0.2	0.0
$\langle {}^1\mathrm{TT}\vert\hat{\mathcal{H}}\vert \mathrm{AC}\rangle$	0.0	0.0	2.4	0.6
$\langle {}^1\mathrm{TT}\vert\hat{\mathcal{H}}\vert \mathrm{CA}\rangle$	0.0	0.0	2.4	0.6
$\langle \mathrm{AC}\vert\hat{\mathcal{H}}\vert \mathrm{S_0S_1}\rangle$	90.7	-8.1	1.6	0.5
$\langle \mathrm{CA}\vert\hat{\mathcal{H}}\vert \mathrm{S_0S_1}\rangle$	-173.1	-25.7	2.2	0.4
$E(\mathrm{CT})$	2.65	3.03	3.87	2.71

5 Triplet state energies

Table S5: Excitation energies (in eV) to the low-lying triplet states computed for the crystal stacked structure at the RASCI and rCAM-B3LYP level with the cc-pVDZ basis set.

state	RASCI	rCAM-B3LYP
$\mathrm{T_1}$	2.08	1.41
$\mathrm{T_2}$	2.10	1.43
$\mathrm{T_3}$	3.56	2.68
$\mathrm{T_4}$	3.56	2.68

6 Thermal fluctuations

Table S6: Electronic couplings (in meV) computed for the crystal structure (cryst.) obtained from 200 snapshots from MD (TM: thermal median) for SEET, TEET, triplet-triplet binding (J) and $^1\mathrm{TT/S_1}$ (TF/SF).

	SEET		TEET		J		TF/SF	
dimer	cryst.	TM	cryst.	TM	cryst.	TM	cryst.	TM
stacked	18.2	27.4	7.2	6.3	7.5	6.4	0.0	65.3
herringbone	30.6	34.1	0.3	0.2	0.0	0.0	0.0	1.1
a-dimer	17.8	19.3	0.0	0.0	0.0	0.0	0.0	0.0
ac-dimer	9.8	10.5	0.0	0.0	0.0	0.0	0.0	0.0

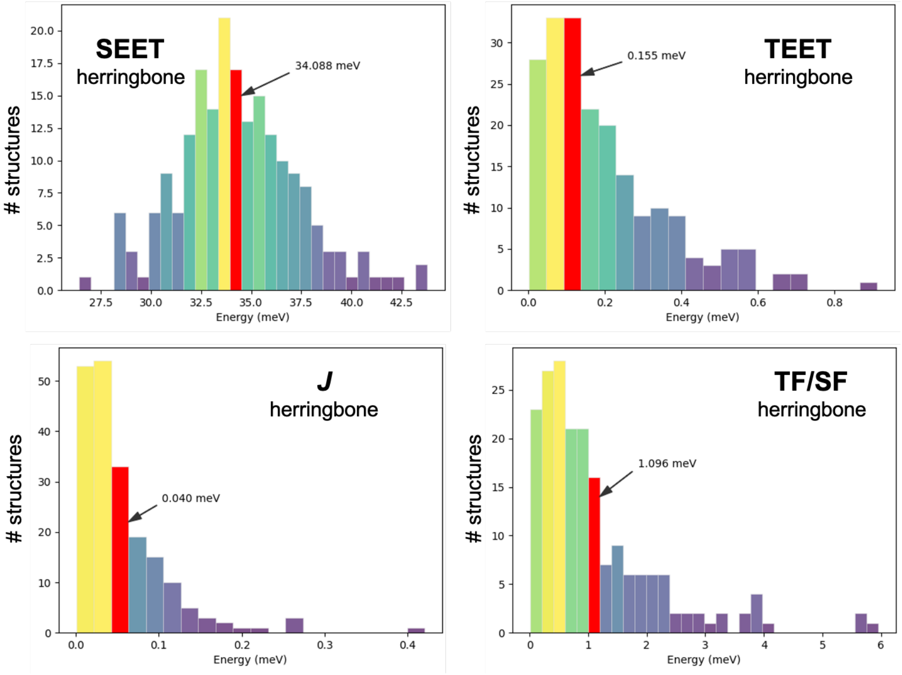

Figure S1: Distribution of SEET, TEET, J, and TF/SF absolute electronic couplings (in meV) in herringbone dimers computed from 200 MD frameworks. Bar colors upon number of structures. Red bar indicates the thermal median (TM).

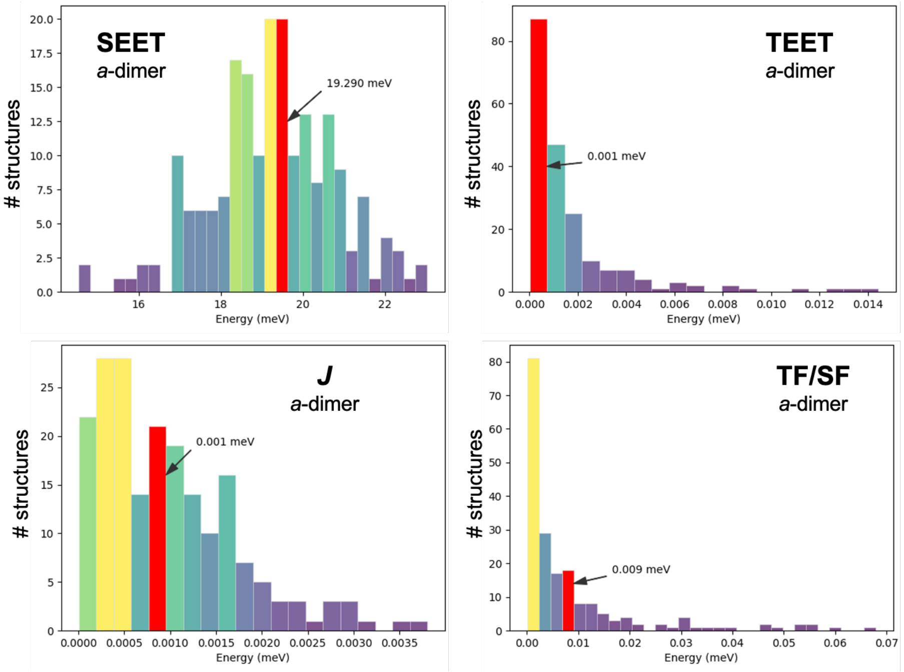

Figure S2: Distribution of SEET, TEET, J, and TF/SF absolute electronic couplings (in meV) in a-dimers computed from 200 MD frameworks. Bar colors upon number of structures. Red bar indicates the thermal median (TM).

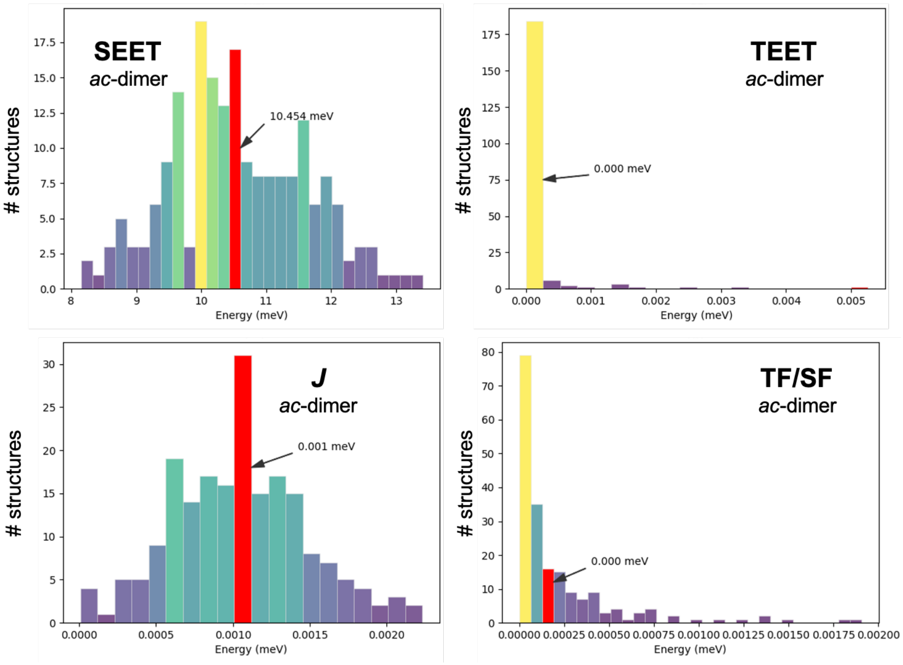

Figure S3: Distribution of SEET, TEET, J, and TF/SF absolute electronic couplings (in meV) in *ac*-dimers computed from 200 MD frameworks. Bar colors upon number of structures. Red bar indicates the thermal median (TM).

7 Structure - coupling analysis

We planned a PCA analysis of structural parameters to find a relationship between structures and electronic couplings. The structural distortions analysed with the thermal fluctuations were not strong enough to be relevant for this analysis. Therefore, a PCA of molecular orbital energies (HOMO-1, HOMO, LUMO and LUMO+1) was performed. This analysis did reveal relevant principal components that have an impact on the electronic couplings.

$$\mathrm{PC1} = 0.50 \cdot E_{\mathrm{HOMO}-1} + 0.48 \cdot E_{\mathrm{HOMO}} - 0.52 \cdot E_{\mathrm{LUMO}} - 0.50 \cdot E_{\mathrm{LUMO}+1} \tag{S.1}$$

$$\mathrm{PC2} = 0.42 \cdot E_{\mathrm{HOMO}-1} + 0.59 \cdot E_{\mathrm{HOMO}} + 0.40 \cdot E_{\mathrm{LUMO}} + 0.56 \cdot E_{\mathrm{LUMO}+1} \tag{S.2}$$

$$\mathrm{PC3} = -0.62 \cdot E_{\mathrm{HOMO}-1} + 0.45 \cdot E_{\mathrm{HOMO}} - 0.53 \cdot E_{\mathrm{LUMO}} + 0.36 \cdot E_{\mathrm{LUMO}+1} \tag{S.3}$$

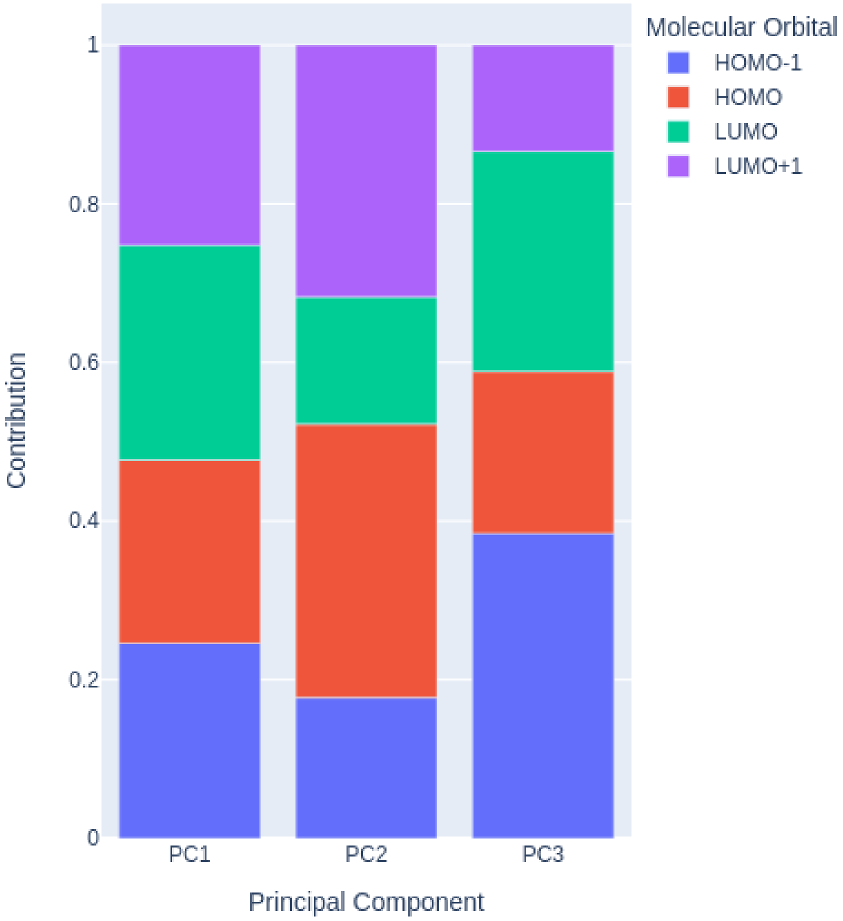

Figure S4: HOMO-1, HOMO, LUMO and LUMO+1 contribution to PC1, PC2 and PC3.

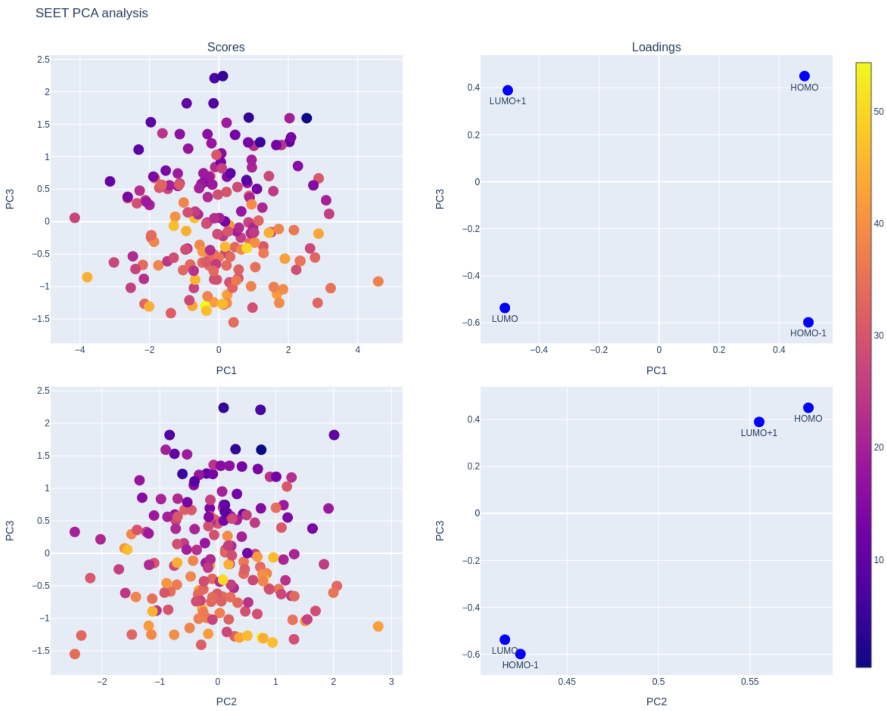

Figure S5: PCA analysis of SEET coupling (in meV) in stacked dimers computed from 200 MD frameworks.

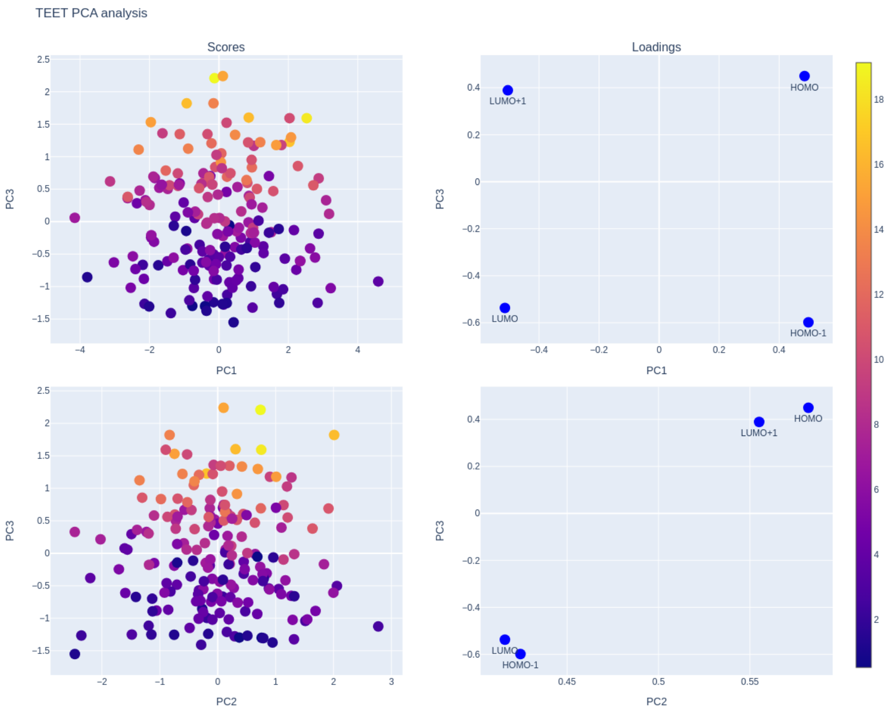

Figure S6: PCA analysis of TEET coupling (in meV) in stacked dimers computed from 200 MD frameworks.

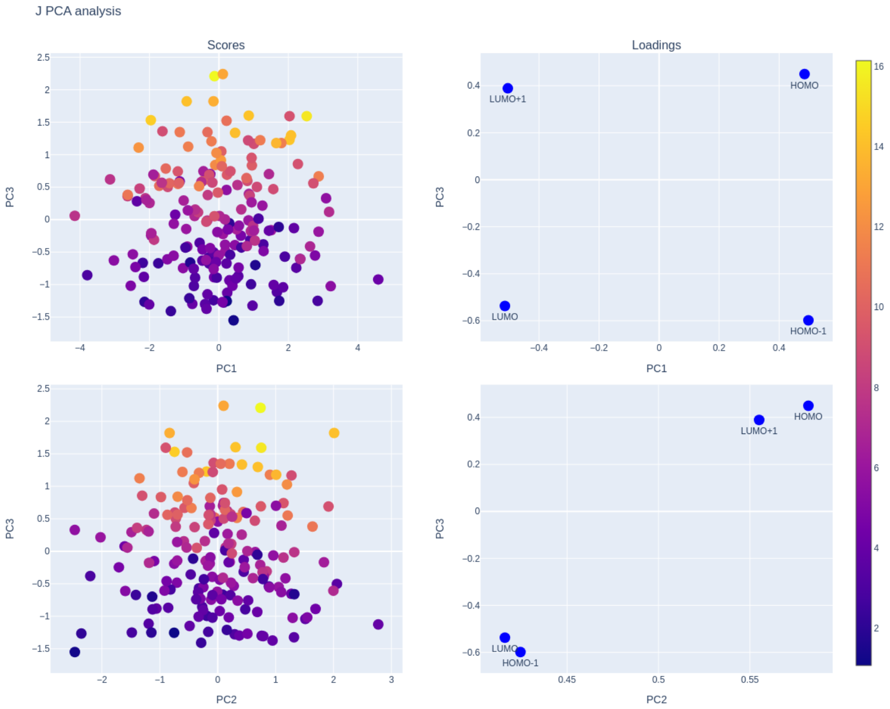

Figure S7: PCA analysis of triplet-triplet binding (J) coupling (in meV) in stacked dimers computed from 200 MD frameworks.

The PCA analysis shows that there is a molecular orbital energies to couplings relationship. In Figure S4 it can be observed that the energies of HOMO-1 and LUMO from one side and HOMO and LUMO+1 from the other side describe the principal component number 3 (PC3). This principal component explains the relationship between SEET values and molecular orbital energies. Similarly, the TEET values and J couplings depend on the molecular orbital energies. However, the relationship between MO energies and SEET couplings is inversely proportional to the relationship with TEET and J couplings.
